# Preoperative parent education and postoperative nurse-led care for boys versus routine hospital care for urethroplasty for hypospadias in Western China: a retrospective study

**DOI:** 10.55730/1300-0144.5811

**Published:** 2024-01-03

**Authors:** Lisi WANG, Zhengdong WANG, Xiaohui MAO, Yingjun FU, Qian LEI, Qin LIAO

**Affiliations:** 1 Department of Pediatric Surgery, West China Hospital, Sichuan University/West China School of Nursing, Sichuan University, Chengdu, Sichuan, China; 2 Department of Pediatric Surgery Nursing, West China Second University Hospital, Sichuan University/West China; 3 School of Nursing, Sichuan University/Key Laboratory of Birth Defects and Related Diseases of Women and Children (Sichuan University), Ministry of Education, Chengdu, Sichuan, China

**Keywords:** Congenital anomaly, hypospadias, postoperative care, urinary obstruction, urinary stent failure, urethroplasty

## Abstract

**Background/aim:**

Urethroplasty is the preferred treatment for hypospadias but is affected by the severity of anomalies, making it a complex procedure with potential postoperative complications. Following surgery, parents receive instructions and recommendations, whether from nurses or physicians, regardless of complication rates. However, nurses play a crucial role in educating caregivers before surgery and providing postoperative care during follow-up. The study aims to assess parents’ knowledge and practices, as well as the frequency of complications in boys who underwent urethroplasty for hypospadias and received postoperative nurse-led care and whose parents received preoperative education against those of boys who underwent urethroplasty under routine hospital care.

**Materials and methods:**

In this retrospective study, Han Chinese boys aged 21–41 months in Western China who underwent urethroplasty for hypospadias were divided into two groups: the NI cohort (n = 103), where they received postoperative nurse-led care and their parents received preoperative education, and the RH cohort (n = 142), where boys underwent routine hospital care.

**Results:**

After urethroplasty, higher numbers of caregivers with satisfactory knowledge (96 (93%) vs. 80 (56%), p < 0.0001) and practice (102 (99%) vs. 132 (93%), p = 0.0276) were reported in the NI cohort compared to the RH cohort. Additionally, a higher number of boys in the RH cohort experienced adverse effects such as moderate bleeding (13 (9%) vs. 1 (1%), p = 0.0052), wound infection (17 (12%) vs. 4 (4%), p = 0.0356), urinary obstruction (35 (25%) vs. 10 (10%), p = 0.0049), burning sensation (47 (33%) vs. 15 (15%), p = 0.0019), and urinary stent fall (32 (23%) vs. 6 (6%), p = 0.0008) compared to those in the NI cohort.

**Conclusion:**

Preoperative instructions enhance caregivers’ knowledge and practices following urethroplasty, while postoperative nurse-led care reduces immediate postoperative complications associated with hypospadias in boys.

## 1. Introduction

Hypospadias is a congenital anomaly characterized by a urethral meatus that opens to the tip along the glands on the ventral surface of the penis, with the meatus shining anywhere from the glands to the perineum [1]. This abnormality can disrupt the typical growth of the penile urethra and foreskin, resulting in penile ventral aspects. This leads to various abnormalities. For instance, the urethral opening may be located along the penile ventral shaft, within the scrotum, or even in the perineum. Additionally, ventral curvature of the penis, known as chordee, may also occur in cases of hypospadias [2].

Hypospadias ranks among the most common urogenital malformations at birth [3]. In China, its prevalence is notably higher, particularly in eastern and southern regions, compared to Western countries [4]. The exact causes of hypospadias remain unclear [3], although both environmental [5] and genetic [4] factors are implicated in its development within the male urinary system.

Urethroplasty is the preferred procedure for hypospadias, with the surgery influenced by the degree of anomalies and the severity of the disorder(s) [6]. The main purpose of surgical interventions is to improve the ability of boys to urinate while standing with a straight flow [7]. However, surgical repairs are intricate, and issues arise in approximately one out of ten children. These issues may include urethro-cutaneous fistula, excessive penile skin, diverticulum, persistent chordee, among others, which can hinder normal penile growth [8].

Nurses play a crucial role in educating caregivers both before surgery and during the postoperative care of patients in follow-up [9]. A quasi-design study conducted before and after surgeries for hypospadias in Egyptian boys revealed that presurgical education for parents reduces the risk of adverse clinical outcomes [10, 11]. Specific preoperative instructions for caregivers can contribute to reducing complications during the follow-up period after hypospadias surgery [10]. In general, parents receive instructions and recommendations after surgery, whether from nurses or physicians, regardless of complication rates. The current standard of care involves educating parents before procedures. To the best of our knowledge, there is no study evaluating postoperative complications specifically associated with Han Chinese boys in Western China. In our routine practice, surgeons often struggle to provide sufficient education to caregivers and child patients who undergo routine hospital care. It is imperative to provide information to parents before any surgery. Ethically, it is not appropriate to withhold information from some parents or caregivers to assess whether this lack of information affects the outcome of surgery. Patient (or caregiver) education about the surgery remains a crucial aspect of any surgical procedure.

The aims of the present retrospective study were to assess parents’ knowledge and practices, as well as the frequencies of complications, in Han Chinese boys aged 21–41 months who underwent urethroplasty for hypospadias and received postoperative nurse-led care, along with preoperative education for their parents. This was compared to those who underwent urethroplasties and received routine hospital care.

## 2. Materials and methods

### 2.1. Inclusion criteria

The study included boys aged 6 months to 4 years who underwent urethroplasty for hypospadias. Only boys and their parents who received instructions for the operation and were available for follow-up care were included in the analysis.

### 2.2. Exclusion criteria

The study excluded boys with recurrent hypospadias and other genitourinary congenital anomalies such as cryptorchidism and vesicoureteral reflux. Additionally, boys with primary or recurrent conditions, which are common reasons for recurrence, were excluded from the analysis. Boys who underwent urethroplasty for hypospad ias but had incomplete data regarding themselves or their caregivers in the institutional records were also excluded from the analysis.

### 2.3. Cohorts

Parents of 103 boys who underwent urethroplasty for hypospadias received preoperative education from nurses and postoperative nurse-led care for the boys (NI cohort). Additionally, 142 boys underwent urethroplasty for hypospadias and received routine hospital care (RH cohort). The boys were divided into two cohorts based on the surgeon’s discretion.

### 2.4. Preoperative education of parents and postoperative nurse-led care

In routine hospital care (the RH cohort), preoperative education was provided by urologists. In contrast, in the NI cohort, preoperative education was administered by nurses in addition to routine hospital care. Preoperative education by urologists or nurses was provided at the hospital. The educational content provided by nurses to parents before surgery remained consistent throughout the study period. Chinese illustrated preoperative instructions for parents and postoperative care for boys were delivered by the nursing staff of the institutes in a standardized manner as follows.

#### 2.4.1. Pain relief

Usually, the nurse encouraged the use of analgesic pumps and oral painkillers. They also educated parents on the importance of these medications in alleviating pain and reducing restlessness during the follow-up period, thus facilitating the healing process.

#### 2.4.2. Improving urinary elimination

The nurse encouraged increased fluid intake after catheter removal and advised drinking liquids on an hourly basis. Parents were instructed to promptly inform the consulting surgeon of any changes in urinary patterns or difficulties voiding.

#### 2.4.3. Reducing anxiety

The nurse encouraged open communication of concerns and allowed parents and boys to ask questions about their condition, the procedures involved, and the recovery process. Using pictures, drawings, and information models, the nurse answered questions calmly and honestly. Additionally, parents and boys were reassured that the defect or surgery would not compromise sexual activity or affect reproductive ability.

#### 2.4.4. Infection prevention

The nurse obtained urine specimens for culture and sensitivities as needed, advising parents to refrain from allowing the boy to straddle toys, play in a sandbox, swim, or engage in rough activities until instructed otherwise by the consulting surgeon. Additionally, the nurse emphasized the importance of applying sterile technique during dressing changes, catheter care, and/or draining urine bags.

### 2.5. Routine hospital care

In the RH cohort, parents received theoretical education. This involved urologists educating them about the disease(s) and the necessity of the invasive procedure before surgery. Following discharge, caregivers were educated on various aspects, including dressing care, diaper management, urinary stent care, medication administration, preventing urinary obstruction, maintaining hygiene, dietary considerations, activity restrictions, and fluid intake.

### 2.6. Outcome measures

Sociodemographic characteristics of caregivers (such as age, education, residence, sex, and number of children), as well as demographic and clinical characteristics of boys, caregivers’ knowledge and related practice assessments, along with postoperative clinical outcomes, were collected and analyzed. All outcome measures were evaluated by the nursing staff of the institute.

#### 2.6.1. Assessment of caregivers’ knowledge

Caregivers’ knowledge was assessed based on a scoring system where a score of 1 was assigned for a correct answer and a score of 0 for a wrong answer or no response. If the total score was less than 50%, caregivers’ knowledge was deemed unsatisfactory; if it was 50% or higher, it was considered satisfactory [3]. This evaluation took place 1 week after surgery. Interviews with caregivers were conducted to assess their understanding of hypospadias surgery using an institutional set of questions designed for caregivers, although these questions have not been published yet.

#### 2.6.2. Assessment of caregivers’ practice

Caregivers’ practice assessment was conducted using a 14-item observational checklist for diaper care [11]. Each item was scored as 1 if the caregiver performed the practice, and 0 if the practice was not performed. If the total score was less than 50%, caregivers’ practice was considered unsatisfactory; if it was 50% or higher, it was deemed satisfactory [3]. This assessment was conducted 1 week after surgery.

#### 2.6.3. Postoperative clinical outcomes

During the 1-month follow-up after urethroplasty, postoperative clinical outcomes including adverse effects, functional outcomes, and cosmetic appearance were evaluated.

### 2.7. Statistical analysis

Statistical analysis was conducted using InStat 3.01 from GraphPad Software Inc. (San Diego, CA, USA). For categorical variables, Fisher’s exact test or the chi-square test for independence (χ2 test; in the four-grid table) was preferred when the total number of cases (n) was greater than or equal to 40 and the theoretical frequency of all grids was greater than or equal to 5. The normality of continuous and ordinal variables was assessed using the Kolmogorov–Smirnov test for Gaussian distributions. For normally distributed continuous and ordinal variables with equal standard deviation (SD), an unpaired *t*-test was utilized. For nonnormally distributed continuous and ordinal variables, the Mann–Whitney test was employed. Correlation analysis among variables, including sociodemographic characteristics of caregivers, demographic and clinical characteristics of boys, caregivers’ knowledge and related practice assessments, and favorable postoperative outcomes, was conducted using Pearson correlation [11]. Results were considered significant if the p-value was less than 0.05 at a 95% confidence interval (CI).

## 3. Results

### 3.1. Study population

From January 15, 2015, to September 1, 2020, a total of 260 boys with hypospadias underwent urethroplasty at the Department of Pediatric Surgery of the West China Hospital, Chengdu, Sichuan, China, Sichuan University, Chengdu, Sichuan, China, West China School of Nursing, Chengdu, Sichuan, China, and the Key Laboratory of Birth Defects and Related Diseases of Women and Children, Chengdu, Sichuan, China. Among them, seven boys had recurrent hypospadias and eight boys had other genitourinary congenital anomalies, thus 15 boys were excluded from the study. Data regarding sociodemographic characteristics of caregivers, demographic and clinical characteristics of boys, caregivers’ knowledge and related practice assessments, and postoperative clinical outcomes of a total of 245 boys from the Western China region who underwent urethroplasty for hypospadias were included in the study. The study flowchart is summarized in Figure.

### 3.2. Sociodemographic characteristics of caregivers

No instances of consanguineous marriage among the parents of any child were reported. Sociodemographic characteristics of caregivers before urethroplasty were comparable between cohorts, with p-values greater than 0.05 for all variables (Table 1).

### 3.3. Demographic and clinical characteristics of boys

Children ranged in age from ≥21 months to ≤41 months at the time of urethroplasty. Demographic and clinical characteristics of boys before urethroplasty were comparable between cohorts, with p-values greater than 0.05 for all variables (Table 2)

### 3.4. Assessment of caregivers’ knowledge

Before urethroplasty, the number of caregivers with satisfactory knowledge was statistically the same in both cohorts. However, after urethroplasty, there was a higher number of caregivers with satisfactory knowledge in both cohorts compared to before urethroplasty. Additionally, after urethroplasty, a higher number of caregivers with satisfactory knowledge were reported in the NI cohort compared to those in the RH cohort. Further details of caregivers’ knowledge assessment are presented in Table 3.

### 3.5. Caregivers’ practice assessment

Before urethroplasty, the number of caregivers with satisfactory practice was statistically the same between both cohorts. After urethroplasty, there were higher numbers of caregivers with satisfactory practice in both cohorts compared to before urethroplasty. However, after urethroplasty, a higher number of caregivers with satisfactory practice were reported in the NI cohort compared to those in the RH cohort. Further details of caregivers’ practice assessment are presented in Table 4.

### 3.6. Postoperative clinical outcomes

#### 3.6.1. Adverse effects

Fever was reported in nearly all boys on the first day after urethroplasty in the postsurgery intensive care unit. Higher numbers of boys in the RH cohort experienced adverse effects such as moderate bleeding, wound infection, urinary obstruction, burning sensation, and urinary stent fall compared to those in the NI cohort. Additionally, a greater total number of adverse events were reported among boys in the RH cohort than those in the NI cohort. Meatal stenosis occurred in 7 (7%) boys in the NI cohort and 17 (12%) boys in the RH cohort, representing a slight increase in boys exposed to meatal stenosis in the RH cohort compared to the NI cohort. Further details of the postoperative short-term and long-term clinical outcomes during the 1-month follow-up after urethroplasty are presented in Table 5.

#### 3.6.2. Functional outcomes and cosmetic appearance

Postoperative functional outcomes and cosmetic appearance during follow-up after urethroplasty were comparable between cohorts, with no statistically significant difference observed (p > 0.05 for all variables, see Table 6). However, there was a slight increase in the number of boys with favorable postoperative functional outcomes and cosmetic appearance in the NI cohort compared to the RH cohort.

#### 3.6.3. Parameter for favorable clinical outcomes

The analysis revealed that higher secondary or higher education of caregivers (p = 0.0412, Pearson correlation), female sex of caregivers (p = 0.0481, Pearson correlation), satisfactory knowledge of caregivers (p = 0.0385, Pearson correlation), and satisfactory practice of caregivers (p = 0.0012, Pearson correlation) were independent parameters associated with favorable clinical outcomes after urethroplasty during the follow-up period. Further details of these parameters for favorable clinical outcomes after urethroplasty in the follow-up period are reported in Table 7.

## 4. Discussion

Before urethroplasty, almost all caregivers had unsatisfactory knowledge and practice of diaper care. Additionally, almost all boys who underwent urethroplasty reported postoperative short-term and long-term clinical complications during the 1-month follow-up period. Hypospadias is a congenital anomaly, and surgery remains the only option for correcting this birth defect. However, even in the hands of the most skilled surgeons, postoperative complications are a regular occurrence [12]. To minimize the occurrence of complications after urethroplasty for hypospadias, comprehensive preoperative education of parents and diligent postoperative care of boys are essential.

After 1 week of urethroplasty, a higher number of caregivers with satisfactory knowledge and practice were reported in the NI cohort compared to before urethroplasty conditions, whereas this improvement was less pronounced in the RH cohort. These findings are consistent with those of the pre- and postsurgical quasidesign study on Egyptian boys [3, 10, 11]. It appears that caregivers who received Chinese-illustrated preoperative instructions showed improved knowledge and practice after urethroplasty [3]. Therefore, preoperative instructions play a crucial role in enhancing the knowledge and practice of caregivers after urethroplasty.

The education of caregivers was reported as an independent parameter for favorable clinical outcomes, which aligns with the findings of pre- and postsurgical quasidesign studies on Egyptian boys [3, 10, 11]. These results highlight the importance of preoperative and postoperative education for caregivers in achieving positive outcomes. This underscores the significance of caregiver education in the Chinese setting as well.

Higher numbers of postoperative adverse effects were reported in the RH cohort compared to the NI cohort, consistent with findings from pre- and postsurgical quasidesign studies on Egyptian boys [3, 10, 11]. The improvement in knowledge and practice of caregivers after urethroplasty in the NI cohort, attributed to preoperative instructions, likely contributed to this difference. Conversely, in the RH cohort, the knowledge and practice of caregivers improved after a few days of follow-up [3]. As a result, immediate postoperative complications were higher when urethroplasty was performed for hypospadias under routine hospital care.

The report of fever in almost all boys on the first day after urethroplasty aligns with findings from pre- and postsurgical quasidesign studies on Egyptian boys [3, 10, 11]. Postoperative mild fever is typically attributed to the inflammatory response triggered by surgery [11]. It is important to recognize that some minor postoperative issues are inherent to the surgical procedure itself. Given these considerations, boys after urethroplasty for hypospadias often require close monitoring in the intensive care unit (ICU).

The report of urinary stent fall as an adverse effect in the RH cohort is consistent with findings from a pre- and postsurgical quasidesign study on Egyptian boys [3], as well as from a prospective study [13], and a cross-sectional study [14]. It is noted that unsatisfactory knowledge and practice of caregivers in the RH cohort likely contributed to the higher incidence of urinary stent fall.

Urinary stent fall was also reported as an adverse effect in the NI cohort. In the current study, children were aged 21 months or older at the time of urethroplasty. At this age, children tend to be hyperactive, making it challenging to retain the urethral stent for the required duration of 7 days with caregivers at home [3]. Therefore, it is suggested that urethroplasty should ideally be performed before 18 months of age to minimize the risk of urinary stent fall after surgery.

The age of boys was not identified as an independent parameter for favorable clinical outcomes after urethroplasty in the follow-up period. However, urethrocutaneous fistulas were reported in a few boys after urethroplasty, with a slight increase observed in the RH cohort. It is noteworthy that urethrocutaneous fistulas are generally reported in older boys after urethroplasty [15, 16]. Older boys are more prone to postoperative complications, particularly urethrocutaneous fistula, due to more frequent penile erections [8]. Additionally, younger boys generally exhibit higher healing abilities compared to older boys [15]. Among these complications, urethrocutaneous fistula is particularly notorious. Considering these factors, performing urethroplasty at a younger age may help mitigate postoperative complications.

Boys in the NI cohort exhibited higher rates of favorable postoperative functional outcomes and cosmetic appearance compared to those in the RH cohort. These findings align with results from a pre- and postsurgical quasidesign study on Egyptian boys [3, 10, 11] and a case-series study [17]. The provision of Chinese illustrated preoperative instructions to parents and postoperative care to boys in the NI cohort likely contributed to decreased postoperative complications and improved favorable postoperative functional outcomes and cosmetic appearance [11]. This underscores the importance of providing comprehensive preoperative instructions to parents and postoperative nurse-led care to boys in ensuring successful outcomes of urethroplasty for hypospadias.

All boys experienced immediate postoperative complications, such as bleeding. However, postoperative nursing of boys and preoperative education of caregivers are not directly related to these very immediate postoperative complications.

The objectives of this study are well-founded and address an important yet underreported area of research. Only one similar study [3, 10, 11] on this issue has been published. The higher incidence of hypospadias in Western China warrants significant research in this area. A standardized approach to care may increase treatment success, lower the number of complications, and decrease the costs of treatment. The approach presented is affordable, practical, and easy to implement. The statistical analysis for the study and the data presented are robust. However, there are limitations to the study, such as a small sample size and its retrospective nature. The lack of a randomized trial significantly affects the quality and significance of the data, but prerandomization was not feasible in this study due to difficulties in controlling confounders. Moreover, while patients were not randomized, both the operator and statistician were blinded. This study does not differentiate between the different types of hypospadias (perineal, scrotal, penile, etc.), which is a weakness and should be acknowledged, as the surgical approach varies depending on the type of hypospadias and is associated with a higher risk of fistula creation/strictures, etc.

## 5. Conclusion

Hypospadias is a birth defect, and surgeries are the only option for correction. However, surgical repairs for hypospadias are complicated and can present challenges. To minimize the occurrence of complications after urethroplasty for hypospadias, preoperative education of parents and postoperative care of boys are essential. Preoperative instructions improve the knowledge and practice of caregivers after urethroplasty. Immediate postoperative complications may be higher if urethroplasty is performed for hypospadias under routine hospital care. Urethroplasty should ideally be performed before 18 months of age to reduce the risk of urinary stent fall and urethrocutaneous fistula after surgery. The data from this study are important and beneficial for healthcare policymakers in designing randomized studies blinded to surgeons. Preoperative instructions to parents and postoperative nurse-led care for boys are necessary components of successful urethroplasty for hypospadias. This study emphasizes the importance of preoperative education and postoperative care for patients.

## Figures and Tables

**Figure:**
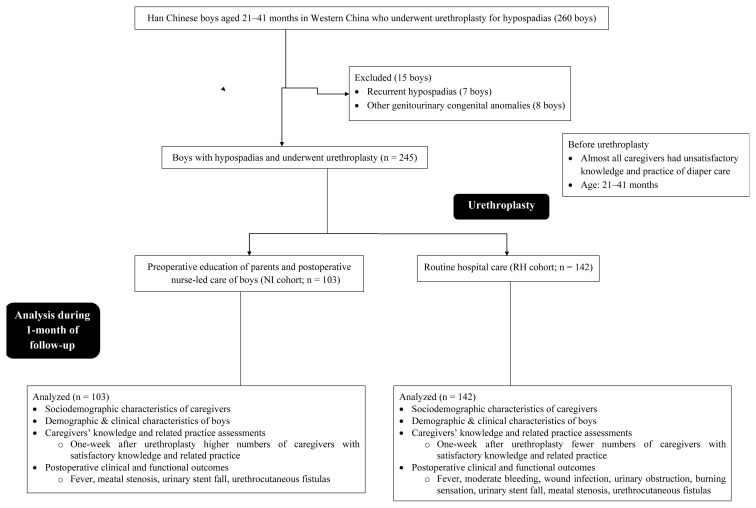
The summary flowchart of the study.

**Table 1:** Sociodemographic characteristics of caregivers before urethroplasty.

Characteristics		Cohorts		Comparisons			
		NI	RH				
Nontreatment intervention(s)		Preoperative education of parents and postoperative nurse-led care of boys	Routine hospital care				
Numbers of caregivers		103	142	*p*-value	Df	Test value	95% CI
Age (years)		33(36–28)	33(38–28)	0.537 (Mann–Whitney test)	N/A	6974.5	N/A
Level of education	Primitive	21(20)	49(35)	0.0859 (χ^2^ test for independence)	3	6.599	N/A
	Secondary	30(29)	40(28)				
	Higher secondary but below graduate	45(44)	46(32)				
	Graduate or more	7(7)	7(5)				
Residence	Rural	21(20)	51(36)	0.0607 (χ^2^ test for independence)	3	7.382	N/A
	Suburban	32(31)	40(28)				
	Urban	35(34)	36(25)				
	Metro city	15(15)	15(11)				
Sex	Female	80(78)	101(71)	0.3156 (χ^2^ test with Yate’s correction)	1	1.007	0.8530 to 1.773
	Male	23(22)	41(39)				
Numbers of children	One	35(34)	45(32)	0.8108 (χ^2^ test with Yate’s correction)	1	0.0573	0.7800 to 1.445
	Two	68(66)	97(68)				

Variable depicted as median (Q3–Q1) or frequencies (percentages).

Df: Degree of freedom, N/A: Not applicable, CI: Confidence interval.

All results were considered significant if the p-value was less than 0.05.

Test value ((χ^2^ value for χ^2^ test, Mann–Whitney U-statistic for Mann–Whitney test).

**Table 2:** Demographic and clinical characteristics of Han Chinese boys before urethroplasty.

Characteristics		Cohorts		Comparisons			
		NI	RH				
Nontreatment intervention(s)		Preoperative education of parents and postoperative nurse-led care of boys	Routine hospital care				
Numbers of boys		103	142	p-value	Df	Test value	95% CI
Age (years)		25.05 ± 1	26.83 ± 0.73	0.1417 (Unpaired t-test)	243	1.474	−0.5985 to 4.161
Family history of hypospadias	Yes	10(10)	10(7)	0.485 (Fisher’s exact test)	N/A	1.21	0.7597 to 1.926
	No	93(90)	132(93)				
Circumcised before hypospadias repair	Yes	4(4)	5(4)	0.9999 (Fisher’s exact test)	N/A	1.059	0.5025 to 2.234
	No	99(96)	137(96)				
History of any other surgeries	Yes	1(1)	1(1)	0.9999 (Fisher’s exact test)	N/A	1.191	0.2955 to 4.802
	No	102(99)	141(99)				

Variable depicted as mean ± standard error of mean or frequencies (percentages).

All results were considered significant if the p-value was less than 0.05.

CI: Confidence interval, N/A: Not applicable.

Test value (t-value for unpaired t-test, relative risk for Fisher’s exact test).

**Table 3:** Assessment of caregivers’ knowledge.

Knowledge assessment NI		Cohorts		Comparisons between cohorts		
	
		RH				

Nontreatment intervention(s)		Preoperative education of parents and postoperative nurse-led care of boys	Routine hospital care			

Numbers of caregivers		103	142	p-value	Relative risk	95% CI

Before urethroplasty						

Unsatisfactory		100(97)	141(99)	0.3125	0.5533	0.3081 to 0.9935

Satisfactory		3(3)	1(1)			
			
After 1 week of urethroplasty						

Unsatisfactory		7(7)	62(44)	<0.0001	0.186	0.09096 to 0.3803

Satisfactory		96(93)	80(56)			

Comparisons between before urethroplasty and after 1 week of urethroplasty	p-value	<0.0001	<0.0001	Not applicable.		

	Relative risk	30.841	56.261			

	95% CI	10.106 to 94.119	8.001 to 395.62			

Variable depicted as frequencies (percentages).

Fisher’s exact test was used for statistical analysis.

All results were considered significant if the p-value was less than 0.05.

Interview with caregivers was performed for evaluation of knowledge about hypospadias surgery (institutional format of questions for caregivers not published yet).

Correct answer: 1 score; wrong answer/no reply: 0 score.

Unsatisfactory: score <50%, satisfactory: score ≥50%.

CI: Confidence interval (using the approximation of Katz.).

**Table 4:** Assessment of caregivers’ practice according to a 14-item observational checklist of diaper care.

Practice assessment		Cohorts		Comparisons		
		NI	RH			
Nontreatment intervention(s)		Preoperative education of parents and postoperative nurse-led care of boys	Routine hospital care			
Numbers of caregivers		103	142	p-value	Test value	95% CI
Before urethroplasty						
Unsatisfactory		68(66)	99(70)	0.6351 (χ^2^ test, degree of freedom:1)	0.2252	0.6678 to 1.233
Satisfactory		35(34)	43(30)			
After 1 week of urethroplasty						
Unsatisfactory		1(1)	10(7)	0.0276 (Fisher’s exact test)	0.2086	0.03199 to 1.360
Satisfactory		102(99)	132(93)			
Comparisons between before urethroplasty and after 1 week of urethroplasty	p-value	<0.0001 (Fisher’s exact test)	<0.0001 (χ^2^ test, degree of freedom:1)	Not applicable.		
	Test value	3.858	3.696			
	95% CI	2.894 to 5.142	2.832 to 4.825			

Variable depicted as frequency (percentages).

All results were considered significant if the p-value was less than 0.05.

Caregivers performed practice: 1 score, caregivers had not performed practice: 0 score.

Unsatisfactory: score <50%, satisfactory: score ≥50%.

CI: Confidence interval (using the approximation of Katz.).

Test value (relative risk for Fisher’s exact test and χ^2^ value for χ^2^ test).

**Table 5:** Postoperative short-term and long-term clinical outcomes during follow-up of 1 month after urethroplasty.

Events	Cohorts		Comparisons		
	NI	RH			
Nontreatment intervention(s)	Preoperative education of parents and postoperative nurse-led care of boys	Routine hospital care			
Numbers of boys	103	142	p-value	Relative risk	95% CI
Bleeding	7(7)	15(11)	0.3698	0.7391	0.3936 to 1.388
Mild bleeding	6(6)	2(1)	0.0722	1.832	1.194 to 2.813
Moderate bleeding	1(1)	13(9)*	0.0052	0.1618	0.02432 to 1.076
Fever	8(8)	17(12)	0.3928	0.7411	0.4102 to 1.339
Wound infection	4(4)	17(12)*	0.0356	0.431	0.1763 to 1.054
Bruising sensation	0(0)	3(2)	0.266	0	-Infinity to Infinity
Wound dehiscence	3(3)	10(7)	0.2477	0.5354	0.1962 to 1.461
Urinary obstruction	10(10)	35(25)*	0.0027	0.4779	0.2712 to 0.8422
Flap necrosis	2(2)	5(4)	0.7022	0.9999	0.2955 to 3.149
Burning sensation	15(15)	47(33)*	0.001	0.5031	0.3158 to 0.8016
Urinary stent fall	6(6)	32(23)*	0.0003	0.337	0.1594 to 0.7124
Urethracutaneous fistula	8(8)	18(13)	0.2938	0.7093	0.3907 to 1.288
Meatal stenosis	7(7)	17(12)	0.1986	0.6714	0.3535 to 1.275
Total	77	231	Not applicable		

Boys had one or more adverse effects(s).

Variable depicted as frequencies (percentages).

Fisher’s exact test was used for statistical analysis.

All results were considered significant if the p-value was less than 0.05.

* Significantly higher than that of the NI cohort.

CI: Confidence interval (using the approximation of Katz.).

**Table 6:** Postoperative functional outcomes and cosmetic appearance during follow-up after urethroplasty.

Outcomes	Cohorts		Comparisons			
	NI	RH				
Nontreatment intervention(s)	Preoperative education of parents and postoperative nurse-led care of boys	Routine hospital care				
Numbers of boys	103	142	*p*-value	df	Test value	95% CI
Urination stream in one direction	80(78)	105(74)	0.6037 (χ^2^ test with Yates correction)	1	0.2694	0.7863 to 1.619
The normal shape of a penis	81(79)	104(73)	0.4122 (χ^2^ test with Yates correction)	1	1.194	0.8243 to 1.730
The normal shape of meatus	78(76)	100(70)	0.4386 (χ^2^ test with Yates correction)	1	0.5998	0.8257 to 1.670
The normal shape of penile skin	81(79)	102(72)	0.2885 (χ^2^ test with Yates correction)	1	1.126	0.8590 to 1.811
Straight penis	92(89)	115(81)	0.1067 (Fisher’s exact test)	N/A	1.535	0.9119 to 2.585

Variable depicted as frequency (percentages).

All results were considered significant if the p-value was less than 0.05.

df: Degree of freedom, N/A: Not applicable, CI: Confidence interval (using the approximation of Katz.).

Test value: χ^2^ value for χ^2^ test and relative risk for Fisher’s exact test.

**Table 7:** Parameter for favorable clinical outcomes after urethroplasty in the follow-up period.

Parameters	Odd ratio	95% CI	**p**-value
Age of caregivers (≥30 years *vs*.<30 years)	0.8521	0.6245–0.8831	0.0821
Education of caregivers (Higher secondary or higher* *vs*. secondary or lower)	1.1211	0.8241–1.2241	0.0412
Sex of caregivers (female* **vs**. male)	1.2231	0.8112–1.3511	0.0481
Age of boy (≥2 years **vs**.<2 years)	0.8211	0.7513–0.9124	0.0561
Circumcised before hypospadias repair (yes vs. no)	0.9521	0.8522–0.9924	0.0512
Knowledge of caregivers (satisfactory* **vs**. unsatisfactory)	1.3321	1.0141–1.5221	0.0385
The practice of caregivers (satisfactory* **vs**. unsatisfactory)	2.4511	1.2511–3.5541	0.0012

CI: Confidence interval.

Pearson’s correlation.

An odd ratio of more than 1 and a p-value less than 0.05 was considered significant.

* Significant parameter for favorable clinical outcomes.

## Data Availability

The datasets used and analyzed during the current study are available from the corresponding author upon reasonable request.

## List of abbreviations

NI cohort: Parents of boys who underwent urethroplasty for hypospadias, and received preoperative education from nurses and ostoperative nurse-led care for their boys
RH cohort: Parents of boys who underwent urethroplasty for hypospadias and received routine hospital care
χ^2^ test: Chi-square test for independence
SD: Standard deviation
Cl: Confidence interval
ICU: Intensive care unit
